# The impact of livestock interventions on nutritional outcomes of children younger than 5 years old and women in Africa: a systematic review and meta-analysis

**DOI:** 10.3389/fnut.2023.1166495

**Published:** 2023-07-06

**Authors:** Josphat Muema, Nyamai Mutono, Stevens Kisaka, Brian Ogoti, Julius Oyugi, Zipporah Bukania, Tewoldeberhan Daniel, Joseph Njuguna, Irene Kimani, Anita Makori, Sylvia Omulo, Erin Boyd, Abdal Monium Osman, Luc Gwenaelle, Christine Jost, SM Thumbi

**Affiliations:** ^1^Institute of Tropical and Infectious Diseases, University of Nairobi, Nairobi, Kenya; ^2^Washington State University Global Health Program–Kenya, Nairobi, Kenya; ^3^Feed the Future Innovation Lab for Animal Health, Washington State University, Pullman, WA, United States; ^4^Center for Epidemiological Modelling and Analysis, University of Nairobi, Nairobi, Kenya; ^5^Centre for Public Health Research, Kenya Medical Research Institute, Nairobi, Kenya; ^6^United Nations Children’s Fund, Nairobi, Kenya; ^7^Food and Agriculture Organization of the United Nations, Nairobi, Kenya; ^8^Paul G. Allen School for Global Health, Washington State University, Pullman, WA, United States; ^9^United States Agency for International Development’s Bureau for Humanitarian Assistance, Washington, DC, United States; ^10^Emergency and Resilience Division, Food and Agriculture Organization of the United Nations, Rome, Italy; ^11^Global Health Support Initiative III, Social Solutions International, Washington, DC, United States; ^12^South African Center for Epidemiological Modelling and Analysis, Stellenbosch, South Africa; ^13^Institute of Immunology and Infection Research, University of Edinburgh, Edinburgh, United Kingdom

**Keywords:** children, women, livestock intervention, undernutrition, nutrition outcome, Africa

## Abstract

**Background:**

Nutrition-sensitive livestock interventions have the potential to improve the nutrition of communities that are dependent on livestock for their livelihoods by increasing the availability and access to animal-source foods. These interventions can also boost household income, improving purchasing power for other foods, as well as enhance determinants of health. However, there is a lack of synthesized empirical evidence of the impact and effect of livestock interventions on diets and human nutritional status in Africa.

**Objective:**

To review evidence of the effectiveness of nutrition-sensitive livestock interventions in improving diets and nutritional status in children younger than 5 years old and in pregnant and lactating women.

**Methods:**

Following PRISMA guidelines, we conducted a systematic review and meta-analysis of published studies reporting on the effect of livestock interventions on maternal and child nutrition in Africa. Data were extracted, synthesized, and summarized qualitatively. Key outcomes were presented in summary tables alongside a narrative summary. Estimation of pooled effects was undertaken for experimental studies with nutritional outcomes of consumption of animal-source foods (ASFs) and minimum dietary diversity (MDD). Fixed effects regression models and pooled effect sizes were computed and reported as odds ratios (ORs) together with their 95% confidence intervals (CI).

**Results:**

After the screening, 29 research papers were included in the review, and of these, only 4 were included in the meta-analysis. We found that nutrition-sensitive livestock interventions have a significant positive impact on the consumption of ASFs for children < 5 years (OR = 5.39; 95% CI: 4.43–6.56) and on the likelihood of meeting minimum dietary diversity (OR = 1.89; 95% CI: 1.51–2.37). Additionally, the impact of livestock interventions on stunting, wasting, and being underweight varied depending on the type of intervention and duration of the program/intervention implementation. Therefore, because of this heterogeneity in reporting metrics, the pooled estimates could not be computed.

**Conclusion:**

Nutrition-sensitive livestock interventions showed a positive effect in increasing the consumption of ASFs, leading to improved dietary diversity. However, the quality of the evidence is low, and therefore, more randomized controlled studies with consistent and similar reporting metrics are needed to increase the evidence base on how nutrition-sensitive livestock interventions affect child growth outcomes.

## Introduction

Children in Africa continue to be at a high risk of undernutrition (stunting, wasting, and being underweight) and this is a serious public health concern in the majority of countries in the continent (1). Despite some progress in combating all forms of malnutrition globally, an estimated two out of every five children under 5 years old and one in every four children under 5 years old in Africa are stunted and affected by wasting, respectively (2). Poverty and malnutrition are responsible for over 250 million children being at risk of not meeting their full development potential later in life (3). Therefore, combating the challenge of undernutrition may contribute to progress in attaining the second and third Sustainable Development Goals (SDG): ending hunger and all forms of malnutrition; and good health and wellbeing. However, for this to happen, there needs to be multi-sectoral strategies and approaches, employing both nutrition-specific and nutrition-sensitive interventions across communities while building more resilient, equitable, and sustainable food systems for improved nutritional outcomes (4–6).

For a majority of rural households in sub-Saharan Africa, agriculture (including livestock) is a key source of livelihood, food, and nutrition security (7, 8). This is supported by previous reviews that have assessed the contribution/impact that general agricultural interventions focusing on home gardening for fruits and vegetables, aquaculture, livestock production, health, cash crops, and biofortified crops have on nutrition (9–20). These reviews have highlighted the growing evidence of the role of nutrition-sensitive agriculture interventions in improving nutrition and documented some pathways through which agriculture can contribute to nutrition. The same can be said of animal-source foods (ASFs), which provide highly bioavailable nutrients that are vital for child growth (21, 22). Livestock and by extension ASFs play a critical role in supporting livelihoods and nutrition security for many communities in sub-Saharan Africa. This role is even more critical for pastoralist communities who inhabit arid and semi-arid areas that have limited potential for crop agriculture due to frequent climatic shocks (23, 24). However, despite the beneficial effects of livestock on child nutrition, livestock keeping is associated with potential adverse effects in women and children due to increased infection and morbidities (25, 26).

Livestock interventions may influence human nutrition through several pathways (27). These include (a) increasing production diversity and consumption of ASFs associated with the ability to meet minimum dietary diversity at the household and individual levels; (b) increasing household income levels through trade in livestock and livestock products, leading to improved household diets (19, 28); (c) women empowerment through increasing women’s socio-economic influence in household decision-making on intra-household food allocation or decisions on food and health expenditure (29–32); and (d) improving productivity through crop-livestock interactions through the provision of manure in the field, and draft power (33). Although livestock interventions are critical as a driver for food and nutrition security, such interventions can impact nutrition both positively and negatively (26). Therefore, when designing and implementing livestock interventions/programs the health impacts need to be well understood and monitored as there may be unintended consequences on social dynamics or the environment (34). Livestock is also associated with negative effects on human health through exposure to zoonotic diseases or proximity to manure and contaminated water or soil (35, 36). However, the nexus between the malnutrition-environment-infection axis is complex, and evidence, particularly on the effect of infectious zoonotic diseases on child nutritional status, is limited (37). Furthermore, livestock interventions may also lead to nutritional risks (25, 38). For example, increased household income through the sale of milk, meat, or eggs may not translate into improved nutrition as a result of household social dynamics (39–41). On the other hand, livestock interventions may also have potentially negative consequences on women’s available time for child care and may increase health and nutritional risks associated with exposure to livestock (19, 42).

As such, livestock interventions targeting dairy production, small livestock husbandry, backyard poultry production, breed improvement, aquaculture, livestock transfer, livestock feeds improvement, and livestock value chain programs, among others, have the potential to improve production diversity, availability, and access to ASFs, dietary diversity at individual and household levels, and impact human nutritional outcomes. However, empirical data on the net contribution of livestock intervention on the nutritional status of vulnerable people in Africa is scant. Therefore, the objective of this review was to synthesize the available evidence on the effectiveness of nutrition-sensitive livestock programs on nutritional outcomes in children under 5 years old and in pregnant and lactating women in Africa. The findings of this study are beneficial for defining current and future program decisions and also for facilitating policy development and advocacy to promote nutrition and food security.

## Materials and methods

### Protocol and registration

This review follows the preferred reporting items for systematic reviews and meta-analysis (PRISMA) guidelines (43). The protocol for this review was prospectively registered on the international prospective register of systematic reviews (PROSPERO) ID: CRD42020203843, https://www.crd.york.ac.uk/prospero/display_record.php?ID=CRD42020203843.

Detailed protocol for this review has also been published elsewhere (44).

#### Definitions

Livestock interventions−for the purposes of this study, livestock interventions were defined as all livestock-related interventions or programs with an objective of increasing production diversity, access to and consumption of animal-source foods (ASFs), and income generation to the households. Such interventions include the provision of livestock feed, provision of animal healthcare, livestock breed improvement, livestock donations, provision of water, provision of shelter, and training/extension services among others.

Livestock−this study loosely defined livestock as all domesticated animals, birds, fish, and insects used as a source of food. This included cattle, camels, goats, sheep, pigs, other small ruminants, poultry/chicken, fish, and bees.

### Criteria for considering studies for the review

#### Study and participant types

This review included studies that evaluated the effect of livestock-oriented programs/interventions on nutritional outcomes in children younger than 5 years old and in pregnant and lactating women in Africa. Nutritional outcomes were defined as anthropometric indices measured by mid-upper arm circumference (MUAC), stunting or height-for-age (HAZ) z-scores, wasting or weight-for-height (WHZ) z-scores, and underweight or weight-for-age (WAZ) z-scores. Furthermore, dietary diversity, micronutrient status (mainly hemoglobin concentrations and prevalence of anemia), and dietary intake of animal-source foods (diet interventions) were also considered.

#### Types of interventions

We targeted studies that used livestock-based interventions with pregnant and lactating mothers and children below 5 years old as intervention groups. The interventions/programs ranged from livestock donations, livestock value chain improvement, livestock ownership compared to not owning any livestock, livestock breed improvement, livestock market participation, provision of livestock inputs and training, supplementation of children’s diets with animal-source foods (ASFs), social behavior change communication (SBCC) intervention to promote the consumption of ASFs, and animal healthcare interventions (vaccination and parasite control).

#### Information source and search strategy

We completed literature searches in major electronic databases including PubMed, Web of Science, and Scopus. The search was conducted by two independent reviewers to identify relevant peer-reviewed publications and online reports. To complement the search, all the reference lists of all studies identified through the database searches and relevant research papers and reports considered were reviewed and the “forward citation” tool in Google Scholar was applied to find research papers that cited these studies. Reference lists of previous systematic reviews conducted on similar study themes were also reviewed.

Search strategies were hinged on the population, intervention, comparison, and outcome (PICO) criteria. These keywords were generated through a preliminary general search in major electronic databases to identify the most used keywords in the publications. Medical Subject Headings (MeSH) search terms were used to identify potential keywords and choose appropriate terms as previously described (44). Boolean operators’ terms “AND,” “OR,” and “NOT” were used to connect the search terms to either narrow or broaden the search. Truncation/wildcard symbol (*) was used for words where variations may be possible (Table 1).

**TABLE 1:** Keywords and search terms used in the database searches.

Indicator	Description
Population	Child OR infant OR pediatric OR “young adult” OR preschool OR pregnant OR woman OR women OR lactating OR breastfeeding OR adolescent OR toddler
Intervention	Trial OR programme OR intervention OR experiment OR supplementation OR implementation OR feed OR consumption OR “Livestock production” OR “livestock ownership” OR pastoral OR livestock OR cattle OR camel OR goat OR sheep OR small ruminant OR poultry OR chicken OR fish OR aquaculture OR fish pod OR pig OR meat OR beef OR mutton OR pork OR dairy OR egg OR honey OR “animal-source food” OR “animal products” OR “foods of animal origin” OR “nutrition sensitive agriculture” OR value chain OR beekeeping OR “animal healthcare” OR water OR shelter OR training OR extension services
Outcome	Nutrition OR nutrition status OR nutrition outcome OR growth OR linear growth OR malnutrition OR undernutrition OR stunting OR wasting OR underweight OR micronutrient OR micronutrient status OR anemia OR hemoglobin OR hemoglobin OR folate OR vitamin OR vitamin A OR vitamin B12 OR iron OR ferritin OR zinc OR calcium OR MUAC OR anthropometric OR height-for-age OR weight-for-height OR weight-for-age OR dietary diversity
Geographical location	Developing countries OR Africa OR Africa, Northern OR Africa South of the Sahara OR sub-Saharan Africa OR Africa, Central OR Africa, Eastern OR Africa, Southern OR Africa, Western OR Algeria OR Angola OR Benin OR Botswana OR Burkina Faso OR Burundi OR Cameroon OR Cape Verde OR Central African Republic OR Chad OR Comoros OR Congo OR “Cote d’Ivoire” OR Djibouti OR “Democratic Republic of the Congo” OR Egypt OR Eritrea OR Ethiopia OR Gabon OR Gambia OR Ghana OR Guinea OR Guinea-Bissau OR Kenya OR Lesotho OR Liberia OR Libya OR Madagascar OR Malawi OR Mali OR Mauritania OR Mauritius OR Morocco OR Mozambique OR Namibia OR Niger OR Nigeria OR Rwanda OR Senegal OR Seychelles OR Sierra Leone OR Somalia OR South Africa OR Sudan OR Swaziland OR Tanzania OR Togo OR Tunisia OR Uganda OR Zambia OR Zimbabwe

MUAC−mid-upper arm circumference.

### Study selection and data abstraction

Article search results were uploaded to Rayyan QCRI^1^ to facilitate collaboration among reviewers during the article selection process. Duplicate articles were removed and the remaining articles were screened by two independent reviewers. Titles and abstracts were screened for eligibility and full-text versions were searched when abstracts did not provide sufficient information to facilitate decision-making.

#### Inclusion and exclusion criteria

Studies were included if they were published in Africa, the study population was children under 5 years old or pregnant and lactating women and involved livestock interventions/programs contributing to the production or consumption of animal-source foods. The outcome of interest in the included studies was nutritional outcomes, including anthropometry [height-for-age z-score, weigh-for-height z-score, weight-for-age z-score, mid-upper arm circumference (MUAC)], micronutrient status, and health-related outcomes. Peer-reviewed articles and online reports published up to 9 December 2021 were included. Studies designed as experimental, quasi-experimental, observational studies, cross-sectional, longitudinal intervention-control comparisons, and randomized field trials were included. Literature reviews, studies conducted in other continents, and studies with crop agriculture interventions, biofortification, home gardening, and irrigation programs were excluded (Table 2).

**TABLE 2:** Inclusion and exclusion criteria used to assess study eligibility.

Criteria	Include	Exclude
Location	Studies conducted in Africa	Studies conducted in other continents
Population	Children under 5 years old, OR pregnant women OR lactating women	
Intervention	Livestock interventions contributing to the production and consumption of animal-source foods (milk, meat, eggs, and fish) and livestock value chains	Crop agriculture Biofortification Home gardening Irrigation programs
Outcome	Nutritional outcomes including anthropometry (weight-for-age z-score, height-for-age z-score, weigh-for-height z-score, MUAC, micronutrient status, and health-related outcomes)	Health outcomes not directly related to nutrition
Publication date	Studies published up to 9 December 2021	
Publication type	Peer-reviewed articles and online reports	Unpublished reports
Study designs	Experimental, quasi-experimental and observational studies, cross-sectional longitudinal intervention-control comparisons, and randomized field trials	Literature reviews
Publication language	English	Other languages

A two-stage screening process was employed in all the retrieved articles from the database searches. First, titles/abstracts were screened by two independent reviewers to check for relevance to the review question. Second, full texts of possible relevant articles were reviewed by two independent reviewers to ascertain if the methods used in the studies selected at stage one adhered to the set inclusion criteria. All articles selected by both reviewers were included for review and data extraction. For articles where there were disagreements between the two reviewers, discussions were carried out with a third reviewer and consensus was sought.

#### Data abstraction

Data were abstracted from relevant articles after a full-text review by the two independent reviewers. Decisions on articles that would be included in the meta-analysis were made independently by each reviewer and discussed between them before arriving at a consensus. Data abstraction variables included study author(s), year and country, study title, study design, study participants and sample size, intervention type, study outcome measured, the effect of the intervention on nutrition, statistical significance, study findings, study limitations, and conclusion.

### Data analysis

Data were synthesized both qualitatively (presenting a summary of key outcomes in the form of summary tables together with a narrative description of the relevant studies) and quantitatively, and key outcomes were presented. The quantitative analysis involved the use of the statistical software Review Manager (RevMan version 5.4.1)^2^ to conduct the meta-analysis. The outcome measures included were consumption of ASFs and minimum dietary diversity. Notably, meta-analysis was not performed on the outcome of nutritional status measured by anthropometric indices (MUAC, stunting, wasting, and being underweight) due to a lack of enough studies reporting similar metrics. The pooled effect of livestock interventions on the consumption of ASFs and meeting MDD was summarized using odds ratios (OR) and their corresponding 95% confidence intervals (CIs). The statistical heterogeneity between studies and its effect on the meta-analysis was determined using the statistical measure of heterogeneity (*I*^2^ statistic) and findings were recorded and interpreted as *I*^2^ statistic (*I*^2^ = 0%: no heterogeneity; *I*^2^ = > 0–≤ 25%: low heterogeneity; *I*^2^ = > 25–≤ 50%: moderate heterogeneity; *I*^2^ = > 50–≤ 75%: high heterogeneity and *I*^2^ = > 75–≤ 100%: very high heterogeneity). Fixed and random effects models were used to estimate the OR (95% CI) based on the level of heterogeneity of the studies included. The results were presented graphically using a forest plot and a summary estimate.

### Bias assessment

Individual studies were assessed for study validity/risk of bias using the grades of recommendations, assessment, development, and evaluation (GRADE) guidelines (45). Studies were scored as either low, medium, or high quality based on five criteria: counterfactual analysis, sample size and power calculations, nutritional outcome assessment, intermediate outcome assessment, and confounding bias assessment. The overall assessment of the risk of bias for each study was determined through a weighted judgment of these established criteria.

## Results

### Selection of studies

**FIGURE 1:**
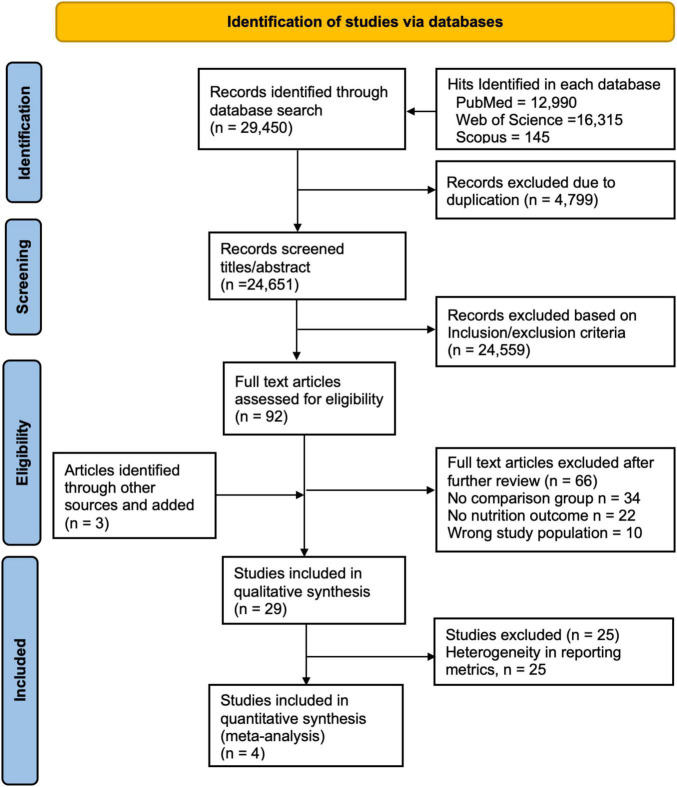
PRISMA flow diagram for inclusion of articles, adapted from Moher et al. (43).

In total, 29,450 articles were retrieved from PubMed (12,990), Web of Science (16,315), and Scopus (145). After excluding 4,799 duplicates, 24,651 articles remained from which 24,559 were excluded after title/abstract review and 66 after full-text review. Three articles were identified through reference list review making a total of 29 articles for which qualitative synthesis was conducted. Of these, four articles were included in the meta-analysis due to the homogeneity of reporting metrics (Figure 1).

**FIGURE 2:**
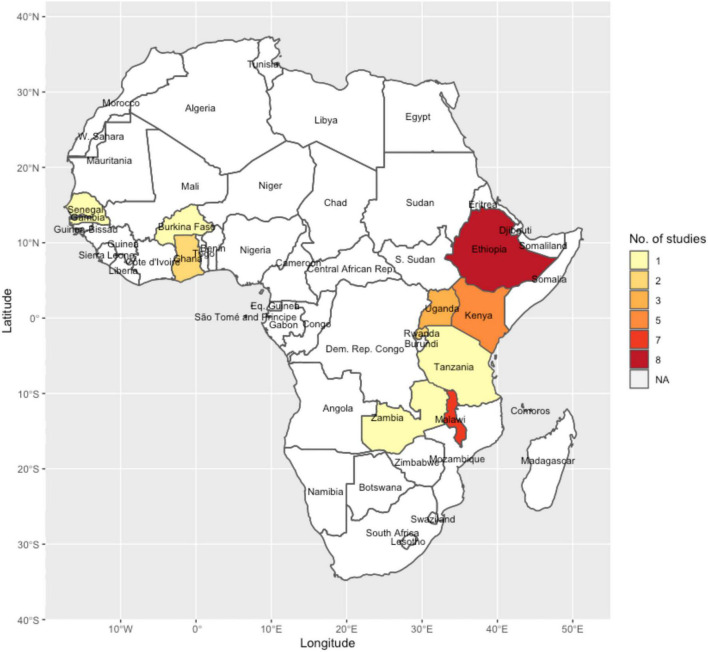
A map of Africa showing the countries where the studies included in the review were conducted and the number of studies in each country.

The studies included in the review were from 10 African countries: Ethiopia (*n* = 8), Malawi (*n* = 7), Kenya (*n* = 5), Uganda (*n* = 3), Rwanda (*n* = 2), Ghana (*n* = 2), Zambia (*n* = 1), Senegal (*n* = 1), Tanzania (*n* = 1), and Burkina Faso (*n* = 1). One study was a regional study involving data from Kenya, Ethiopia, and Uganda (Figure 2).

### Summary of study characteristics and their evidence

The studies are described based on nutritional outcomes, study design, and type of livestock intervention. Based on nutritional outcomes, the studies were classified as either addressing dietary diversity, consumption of animal-source foods (ASFs), hemoglobin concentration or prevalence of anemia, stunting or HAZ z-scores, wasting or WHZ z-scores, and underweight or WAZ z-scores. Some studies had multiple outcomes. For the nutritional outcomes measured, dietary diversity was reported by 9% (*n* = 5) of the studies (46–50), consumption of ASFs by 18% (*n* = 10) of the studies (27, 46, 50–57), hemoglobin concentration and prevalence of anemia by 7% (*n* = 4) of the studies (51, 58–60), stunting or HAZ z-scores by 33% (*n* = 18) of the studies (27, 46–48, 51–53, 55–57, 61–68), wasting or WHZ z-scores by 15% (*n* = 8) of the studies (27, 46, 47, 51, 55–57, 61), and 18% (*n* = 10) reported on underweight or WAZ z-scores (27, 46–48, 53, 60, 61, 68–70) Based on study design, of the 29 studies reviewed, 14% (*n* = 4) were livestock oriented impact evaluations, 4% (*n* = 1) evaluated dairy sensitive value chains, 41% (*n* = 12) were observational studies, and 41% (*n* = 12) were experimental studies (Supplementary Table 1).

Of these studies, 24% (*n* = 7) focused on the provision of ASFs in diets, 14% (*n* = 4) were livestock donation interventions, 14% (*n* = 4) were on ownership of dairy cows and association with nutritional outcomes, 10% (*n* = 3) were on livestock ownership and child nutrition and health outcomes, 10% (*n* = 3) were on poultry interventions, and 7% (*n* = 2) were on the consumption of ASFs. In addition, 3% (*n* = 1) of the studies were on nutrition-sensitive dairy value chains, 3% (*n* = 1) on fish farming, 3% (*n* = 1) on analysis of national datasets, 3% (*n* = 1) on milk market participation, 3% (*n* = 1) on animal health intervention, and 3% (*n* = 1) reported on an SBCC intervention on consumption of ASFs.

### Meta-analysis

Of the four research papers included in the meta-analysis, the majority (*n* = 3) were on poultry-related livestock interventions. However, some also included an additional component of training on either health and nutritional behavior change communication or livestock husbandry training (Table 3).

**TABLE 3:** Description of four studies included in the meta-analysis based on country, study design, and intervention components.

Study, country (reference)	Study design	Intervention component			
		**Inputs**		**Training**	
		**Poultry/eggs**	**Livestock**	**Health/nutrition BCC**	**Livestock husbandry**
(48) Ghana	cRCT	X		X	X
(50) Rwanda	cRCT		X	X	
(56) Malawi	RCT	X		X	
(49) Malawi	RCT	X			

cRCT, cluster randomized controlled trial; BCC, behavior change communication.

### Pooled effect estimates

**FIGURE 3:**
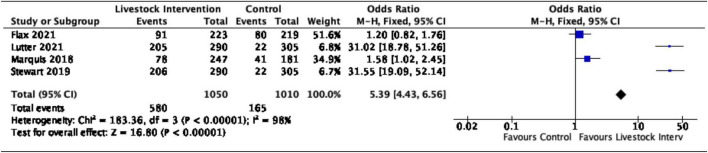
Effects of nutrition-sensitive livestock interventions on the consumption of ASFs in children < 5 years of age.

The pooled effects of nutrition-sensitive livestock interventions on the consumption of ASFs and MDD outcomes in children < 5 years of age were estimated. Generally, nutrition-sensitive livestock interventions were associated with increased odds of consumption of ASFs, (OR = 5.39, 95% CI = 4.43–6.56). However, substantial heterogeneity was detected between the studies (*I*^2^ = 98%, *p* = < 0.00001), as shown in Figure 3.

**FIGURE 4:**
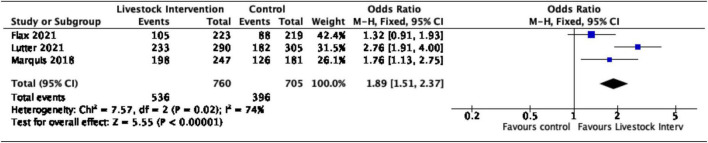
Effect of nutrition-sensitive livestock interventions on minimum dietary diversity in children < 5 years of age.

Additionally, nutrition-sensitive livestock interventions were associated with an 89% increase in the likelihood of children aged < 5 years attaining minimum dietary diversity (OR = 1.89, 95% CI = 1.51–2.37). Moderate heterogeneity was reported for this sub-group with the *I*^2^ proportion being 74% (Figure 4).

### Rating quality of evidence

Based on the GRADE quality of evidence assessment approach, the overall quality of evidence of this review was rated low, mainly due to limitations of performance, inconsistency, and selection biases; for more details see Table 4.

**TABLE 4:** GRADE rating of the quality of evidence.

Certainty assessment							No of participants		Effect		Certainty	Importance
**No of studies**	**Study design**	**Risk of bias**	**Inconsistency**	**Indirectness**	**Imprecision**	**Other considerations**	**Livestock interventions**	**Control**	**Relative (95% CI)**	**Absolute (95% CI)**		
**ASFs consumption (follow-up: mean 12 months)**												
4	Randomized trials	Not serious	Serious^a^	Not serious	Serious^b^	Not serious	580/1,050 (55.2%)	165/1,010 (16.3%)	**OR 5.39** (4.43 to 6.56)	**349 more per 1,000** (from 300 more to 398 more)	⊕⊕○○ low	Important
**Minimum dietary diversity (MDD) (follow-up: mean 12 months)**												
3	Randomized trials	Not serious	Serious^c^	Not serious	Serious^b^	Not serious	536/760 (70.5%)	396/705 (56.2%)	**OR 1.89** (1.51 to 2.37)	**146 more per 1,000** (from 98 more to 191 more)	⊕⊕○○ low	Important

CI, confidence interval; OR, odds ratio. ^*a*^High levels of heterogeneity of the study’s results. ^*b*^Small sample sizes and wide confidence intervals. ^*c*^Moderate level of heterogeneity in the study’s results. The bold values indicate to the corresponding absolute effect estimates. ⊕⊕○○Means low level of certainty.

## Discussion

This review synthesized existing evidence of the effect of nutrition-sensitive livestock-oriented programs/interventions on diet and nutritional outcomes in children below 5 years old and in pregnant and lactating women in the African setting. We synthesized the findings of peer-reviewed articles from three databases. The analysis of evidence related to the association of livestock interventions/programs with the nutritional status of women and children showed that despite the drawbacks associated with keeping livestock, such as a risk factor for disease and mortality in children (74), livestock interventions have positive dietary benefits.

Evidence on how livestock programs/interventions influence child nutritional outcomes has increased and improved since the reviews reported by Grace et al. (11) and Leroy and Frongillo (12). In 2018, Ruel et al. (19) synthesized evidence on the linkages between nutrition-sensitive agriculture programs and nutritional outcomes. However, their review is different from ours because the former focused on general agriculture interventions including homestead food production systems, home vegetable gardens, biofortified crops, livestock, and irrigation projects and their effect on nutrition in the general population. Our review focused mainly on livestock-oriented interventions and their effect on nutrition in children under 5 years old and/or pregnant and lactating women specifically in Africa.

Based on our evidence synthesis, a sizeable percentage of articles showed that livestock interventions improved access to and consumption of nutrient-dense animal-source foods (27, 46, 50–57), attaining minimum dietary diversity (46–50), hemoglobin concentration, and prevalence of anemia (51, 58–60). Additionally, some livestock interventions improved children’s stunting or height-for-age (HAZ) z-scores (27, 46–48, 51–53, 55–57, 61–68), wasting or weight-for-height (WHZ) z-scores (27, 46, 47, 51, 55–57, 61), and underweight or weight-for-age (WAZ) z-scores (48, 61, 68, 69), which are indicators of chronic and acute nutritional status in children. This positive effect is because livestock and livestock products are a source of essential, nutrient-dense, and highly bio-available ASFs and are a source of household income through sales of livestock and livestock products, which translates to improved nutritional status among women and children in underserved and vulnerable populations.

Overall, effects were reported on children’s diets including consumption of animal-source foods, meeting minimum dietary diversity. Linear growth and better HAZ z-scores were reported specifically for milk consumption interventions. However, the effect on stunting, wasting, and being underweight varied, with some studies reporting effects on WAZ and WHZ z-score but not on HAZ and vice-versa depending on the type of intervention. For example, a livestock transfer program in Rwanda, the distribution of small animals through revolving funds in Malawi, and the establishment of small-scale egg production centers in Zambia documented positive effects on the consumption of animal-source foods (46, 51, 52). Overall, this review reported successes in increasing production diversity and consumption of animal-source foods. The documented effect of interventions evaluated was mainly the improved access to and consumption of nutrient-dense animal-source foods and improved dietary diversity. However, the effect reported on nutritional status measured by height-for-age z-scores (stunting), weight-for-height z-scores (wasting), and weight-for-age z-scores (underweight) was either weak or not present at all. Similarly, evidence of the impact on micronutrient status was also uncommon with only one study reporting an effect on Hb concentrations in children (58). Marquis et al. (48) assessed the impact of a livestock intervention involving the donation of improved chicken for egg production, provision of inputs, and husbandry training on diet diversity in Ghana. It was found that children in the intervention group met minimum dietary diversity and had higher HAZ, and WAZ z-scores (48). An animal health intervention in rural Kenya increased the consumption of ASFs and improved child growth. This intervention involved vaccination of chicken against Newcastle disease and parasite control while the control group received only parasite control. The intervention increased both HAZ and WHZ z-scores in the intervention group relative to the control group (57).

Similarly, a few studies showed that livestock interventions improved child HB concentrations thus reducing anemia in children. In rural Senegal, a cluster randomized controlled trial (58) tested the effect of using a dairy value chain to distribute micronutrient-fortified yogurt to improve hemoglobin levels (Hb) and reduce iron deficiency anemia among children aged 24–59 months and showed improved Hb concentrations and reduced prevalence of anemia. In eastern Ethiopia, the consumption of camel milk by pastoralist communities was associated with a lower prevalence of anemia when compared to cow milk consumption (72). Additionally, a small animal revolving funds intervention program in Malawi yielded a decrease in the prevalence of anemia in pregnant women and preschool children (51). Notably, this very program was implemented as an integrated package that included iron supplementation and malaria control, hence it was difficult to attribute the effect to a specific component of the program.

Generally, livestock ownership is associated with increased consumption of animal-source foods such as milk, meat, and eggs. Milk consumption was positively associated with child linear growth, particularly in households that owned milking animals (75). The majority of the livestock-oriented observational studies reviewed showed an association between livestock ownership, consumption of animal-source foods, household, or individual dietary diversity, and in some cases child nutritional status. However, these associations were context-specific, and several effect modifiers on the association between livestock ownership and consumption of ASFs and child nutritional outcomes were identified. These included market access, socioeconomic status, income, number of livestock owned, livestock diseases, and food security status. Market access was the main effect modifier on the effect of livestock ownership and consumption of animal-source foods and nutritional outcomes of children. This suggests that milk market development and access to milk markets can be an alternative to household livestock ownership (53). To support this, a study conducted in Nepal reported that food markets regulate dietary intake and households with better access to markets are less vulnerable to seasonal variations in dietary intake and nutritional status (76).

The studies on dairy cow ownership showed that dairy production is associated with increased milk consumption and better child nutritional outcomes in Ethiopia, Uganda, Tanzania, and Kenya. In Ethiopia, specifically, cow ownership was also associated with a lower prevalence of childhood stunting, and increased linear growth (53). However, this association was context-specific and dependent on market access. No association was observed between cow ownership and stunting in households with good access to local markets. In Uganda, cow ownership was associated with increased milk consumption and reduced stunting (HAZ) but not with underweight (WAZ) and wasting (WHZ). Even then, the reduced stunting was only seen in households with large farms (27). In Tanzania, dairy production predicted reduced levels of stunting, wasting, and being underweight although this association was only observed among poorer households (61). In Kenya, child nutritional outcomes among children from dairy farmers and dairy customers were compared to those from rural households not practicing dairy farming. It was found that milk consumption was a good predictor of better nutritional outcomes for all levels of stunting, wasting, and being underweight for dairy farmers and dairy customers with the same household income compared to households not practicing dairy farming (70). A pathway analysis of the relationship between ownership of improved dairy cow breeds and child nutritional outcomes in Uganda showed that milk consumption was associated with improved HAZ z-scores (27).

Diet interventions involving the consumption of ASFs showed improved nutritional outcomes. Long et al. (65) evaluated a 5-month comparison feeding intervention of an animal-source foods program on toddler growth in rural Kenya. The program involved the provision of plain porridge (no ASF), meat porridge, and milk porridge (65). It was found that linear growth was significantly greater for the milk group than the meat group and plain porridge group although the small sample size and short follow-up period limited the clarity of the results. In addition, Argaw et al. (66) evaluated a fish oil supplementation intervention on linear growth, morbidity, and systemic inflammation among children aged 6–24 months in Ethiopia. Surprisingly, no significant effect of fish oil supplementation on linear growth was found (66). Furthermore, when Lutter et al. (49) assessed the impact of a 6-month egg complementary feeding intervention in Malawi on energy intake and dietary diversity among children aged 6–9 months, there was an improvement in usual energy intake and dietary diversity in the intervention group compared to the control group (49). Bierut et al. (67) examined the effect of daily supplementation of bovine colostrum/egg in Malawi compared to isoenergetic corn/soy flour on linear growth faltering among children aged 9–12 months. The intervention reduced growth faltering among children in the intervention group compared to those in the control group (67). Caswell et al. (54) assessed the impact of an egg intervention on nutrient adequacy among young Malawian children and found that the intervention resulted in increased intakes of protein and several micronutrients.

Not all studies reported a positive or beneficial relationship between livestock interventions and nutritional outcomes in women and children. Many studies reported no significant differences between intervention and control groups. A dairy goat donation program in Ethiopia did not find any differences in the consumption of animal-source foods, which the authors attributed to the evaluation being conducted too early to detect any accrued improvements (71). Similarly, no effect on child HAZ z-scores was demonstrated in a livestock transfer project in Rwanda (46). In Malawi, Prado et al. (73) and Stewart et al. (56) examined the effect of an egg intervention on child development scores and child linear growth, respectively, among children participating in a project. The project involved the provision of one egg per day to children aged 6–15 months coupled with guidance on hygiene and handwashing during food preparation to mothers of both the intervention and control groups. No significant difference was observed between the intervention and control groups on child development scores (73). Similarly, no significant intervention effect on height-for-age, weight-for-age, and weight-for-height z-scores was observed (56).

Generally, the design and methods of studies on the effect of nutrition-sensitive livestock interventions have improved. This is attributed to the adoption of experimental and quasi-experimental designs, coupled with clearer objectives and better control study arms. However, the greatest limitations that hinder the generalizability of findings from these studies remain as small sample sizes and shorter periods of intervention implementation. This is in addition to the complexity of the majority of the programs being integrated, which makes it difficult to assess the effect of individual program components on nutritional outcomes. The small sample sizes and short periods of program implementation might explain the lack of effect of the interventions on height–for–age (HAZ) z-scores in some of the studies, as stunting is a long-term measure. The quality of the livestock-oriented observational studies reviewed was varied. There is a general improvement in quality with recent studies using better statistical methods, and well-defined age groups of study participants as they assessed nutritional status indicators. However, these observational studies used a cross-sectional design making it impossible to infer causality. Additionally, some studies used nationally representative datasets such as DHS, which could have large variations in some observed characteristics.

Important to note is the increase in the number of experimental studies looking at the effects of livestock interventions on child nutritional outcomes, especially the randomized controlled trials. Of the 12 randomized controlled trials (RCTs) reviewed, 7 were on the provision of animal-source foods in diets (49, 54, 56, 65–67, 73); 3 involved poultry interventions coupled with a training program (48, 55, 68); 1 was on SBCC on the consumption of animal-source foods (50); and 1 was on vaccination of chicken against Newcastle disease (77). All these studies likely presented good-quality evidence since they were randomized controlled trials with counterfactual analysis. These interventions were either implemented alone or incorporated nutrition and health behavior change communication (SBCC) strategies. Coupled with this, the analysis methods used were either baseline and end-line comparisons or regressions to determine the treatment effect for intervention-control comparisons.

Women empowerment in decision-making and engagement in livestock programs is a key pathway from livestock to improved child nutrition (31, 78). Women have been shown to play a significant role in household nutrition (79–81). Thus, livestock-oriented nutrition-sensitive programs should target animals or animal products that women have access to and control so as to ensure maximum benefits for women’s and children’s nutrition (82).

Although infection/morbidity was not considered as an outcome in the present review, livestock interventions, particularly keeping livestock, may be a significant risk factor for increased risk of disease and thus, could negatively influence nutritional outcomes in women and children (36, 38, 62). As such, much as livestock ownership has a positive association with the consumption of nutrient-dense animal-source foods and better nutritional outcomes, it also predicts negative health consequences due to increased exposure to animal waste. In Ethiopia, Headey and Hirvonen (62) found a positive association between poultry ownership and child height-for-age z scores. However, the practice of corralling poultry in household dwellings overnight was negatively associated with child height-for-age z scores. This is possibly due to increased children’s exposure to chicken feces, leading to an increased risk of infection (62).

In rural Kenya, a one-year cohort study that followed up children below 5 years old found no association between livestock ownership and child growth. The authors attributed this to a potentially high disease burden among the children (64). However, this study could not determine whether the disease burden was due to the actual transmission of diseases between livestock and humans or the impact of livestock diseases on household economies. In Ghana, it was observed that children from households owning livestock were less likely to have anemia compared to those from non-livestock-owning households. Additionally, livestock ownership was not associated with child morbidity (59).

The effect of livestock ownership on child morbidity is varied, with some studies hypothesizing that livestock ownership may indirectly be associated with negative effects, particularly morbidity due to exposure to animal feces (62). This means that hygiene might be an important mediating factor linking livestock ownership to child growth. Future reviews on this topic should incorporate infection status and morbidity for both women and children, especially in African settings. Consequently, since livestock is hypothesized to expose children to animal feces, especially chicken and animal diseases, there is a need to integrate such programs with sanitation, and hygiene (WASH) plans. Furthermore, studies on fecal pathogen pathways should be studied when assessing nutrition-based interventions. In addition, there is increasing evidence of the negative impacts of livestock on child gut health and child nutrition (25). Exposure to enteric pathogens leads to chronic infection of the intestines and inflammation of the gut leading to dietary deficits. To ensure a comprehensive assessment of the effects of livestock on nutrition and health outcomes, there is an urgent need to include poor gut health as an immediate determinant of child undernutrition, hence effectively expanding the UNICEF framework to include inadequate dietary intake, disease, and poor gut health as immediate causes of malnutrition (25).

The pathways from livestock interventions to improved nutritional outcomes could be mediated by many factors, including household incomes, access to markets, and seasonality. In Ethiopia, higher levels of milk production, household income, dietary diversity, and child nutritional status were observed in milk market participating households compared to non-participating households (47). However, despite the significant differences in household milk production between milk market participating households and non-participant households, no significant differences were observed in the consumption of ASFs generally and milk consumption specifically. Therefore, the better dietary diversity and nutritional status of children in milk market participating households could potentially be attributed to increased household income.

Incorporating a training component or a social behavior change communication (SBCC) component in nutrition-sensitive livestock programs could be beneficial in improving nutritional outcomes. In Rwanda, Flax et al. (50) investigated the effect of a social behavior change communication intervention promoting the consumption of ASFs on maternal ASFs knowledge, child milk consumption, and dietary diversity among beneficiaries of a livestock transfer program (50). The intervention was associated with increased maternal knowledge of ASFs and child milk consumption. However, there were no significant differences between the intervention and control groups on diet diversity. Similarly, the SBCC intervention did not influence household milk retention or the decision to sell milk, depicting that nutritional education alone is not enough to change nutritional outcomes in households with poor food security. Similarly, no differences in anthropometric indices (HAZ and WAZ z-scores) between the intervention groups were observed in Malawi when Passarelli et al. (68) assessed the impact of a poultry intervention with or without an additional nutrition BCC component on child nutritional status (68). Further, in Burkina Faso, McKune et al. (55) evaluated the effect of livestock intervention (chicken gifting) and a culturally tailored behavior change package on child egg consumption and nutritional status. The intervention involved two components, full intervention (gifting chicken + nutrition BCC) and exclusive Nutrition BCC. Both interventions significantly increased egg consumption compared to the control group while full intervention significantly decreased wasting and children being underweight (55).

Social behavior change communication interventions had an impact on the increased consumption of ASFs. However, this consumption was influenced by production and food security situation. For effective and impactful SBCC interventions, they could be tailored with the objective to increase production diversity. Furthermore, these interventions should aim to influence decision-making around the retention of animal-source products for home consumption. Finally, SBCC interventions should target influencing how proceeds from the sale of animal-source products could be used for household nutrition.

Although promising, livestock programs for improved nutritional outcomes still need more evidence to be able to confirm causal inference (19). For example, of the 29 articles included in the evidence synthesis, 12 were randomized controlled trials reporting on varied livestock interventions/programs and nutritional outcomes. The increase in the number of randomized trials on nutrition-sensitive livestock programs is encouraging and will help elucidate empirical evidence on the influence of livestock interventions/programs on nutritional outcomes. However, livestock interventions/programs are by nature integrated, complex, and involve multiple outcomes that need to be taken into account when designing such trials. A recent research paper by Leroy et al. (83) provided guidance on how to strengthen causal inference from randomized controlled trials of complex interventions to ensure such trials are conducted adhering to the highest scientific standards. Such guidelines will be critical for future nutrition-sensitive livestock programs in providing the much-needed empirical evidence of their effectiveness in improving nutritional outcomes.

Our review is subject to some limitations that ought to be taken into account when interpreting the study’s findings. One limitation is that we synthesized evidence from heterogenous study designs and outcome variables that potentially affect some of the research conclusions. The second weakness of the review is that we synthesized evidence based on the direction of the association and focused on the positive effects of livestock interventions and did not consider infection status and morbidity outcomes, and therefore the review did not provide a holistic approach to the effect of livestock interventions on health and nutrition. The other potential limitation is that we only had a very small (four) number of studies that were included in the meta-analysis and from which pooled effect sizes were calculated, which might have reduced the precision of our estimates. This was because of heterogeneity in reporting metrics of the studies included in the review. Furthermore, we left out other important outcomes such as women empowerment and seasonality of malnutrition in our review, which could have provided a clearer picture of the pathways from livestock interventions to improved nutritional status.

Despite the aforementioned limitations, our review has several strengths that render the study’s findings useful and contributes to the body of evidence in this field. The computation of pooled effect sizes on the impacts of livestock interventions on nutritional outcomes is the first step in providing the much-needed evidence of the impact of nutrition-sensitive livestock interventions/programs on nutritional outcomes for vulnerable communities. Second, the focus of our study in the less-studied African continent provides evidence for governments and development partners for decision decision-making. Furthermore, focusing on livestock interventions provides an excellent opportunity to elucidate evidence of the net contribution of livestock to human nutritional outcomes and could provide evidence for a policy shift in nutrition-sensitive programs, particularly for livestock-dependent communities.

## Conclusion

Generally, our review found considerable evidence underscoring the beneficial effects of nutrition-sensitive livestock interventions on the nutritional outcomes of women and children. This was mainly through increased consumption of ASFs, improved dietary diversity, and, in some instances, child nutritional status (stunting, wasting, and being underweight). Substantial heterogeneity in reporting metrics across studies was detected, which limited the number of studies and outcomes that could be included in the computation of pooled effect sizes. Overall, despite the growing number of studies on this subject, the quality of the evidence is still low, particularly in the African setting.

### Program and policy recommendations

Despite the growing body of proof of the link between nutrition-sensitive livestock interventions on nutritional outcomes, there is still a paucity of empirical evidence and consensus on the effectiveness and cost-effectiveness of these programs on nutritional outcomes. For example, none of the studies reviewed carried out an economic evaluation of the interventions. In addition, much as economic evaluation studies of agriculture, nutrition, and health projects are gaining prominence with the development of guidelines by the Action Against Hunger (ACF) (84), there is an urgent need for data on the cost-effectiveness of livestock-oriented nutrition-sensitive interventions on nutritional outcomes. Future studies need to incorporate an economic evaluation component to determine the cost-effectiveness of nutrition-sensitive livestock interventions in improving nutritional outcomes in women and children.

Although livestock interventions have shown the potential to improve children’s diets through the consumption of nutrient-dense ASFs and improving dietary diversity, more research is required to understand the risks posed by animal rearing on child nutrition, particularly on morbidity, disease, and gut health in the African setting (25, 74).

Most of the programs reviewed were implemented based on donor funding cycles that were limited to 1–2 years on average with no scale-up strategies being reported. This short-term implementation duration may have masked the true magnitude of the effects of these interventions. There is, therefore, a need for longer-term interventions with scale-up strategies to meaningfully influence nutritional outcomes, such as stunting. Better-designed randomized controlled trials are required to better determine the effectiveness of livestock interventions on nutrition outcomes of stunting, wasting, and being underweight. Such studies should be designed from the onset with these nutritional objectives and should be powered to determine treatment effects on stunting, wasting, and being underweight. There is also a need for studies with designs that allow causal inferences on the observed effects.

Furthermore, livestock productivity and subsequent child nutritional outcomes are prone to seasonal variations and climatic shocks. This means that nutrition-sensitive livestock interventions that prevent seasonal variation in child nutritional outcomes can potentially improve nutritional outcomes. However, evidence on the effect of livestock programs that address the seasonality of malnutrition is limited. There is, therefore, need for studies that explore the effect of livestock interventions on nutrition during emergencies or climatic shocks such as drought. Finally, since several factors have been confirmed to modify the effect of livestock interventions on child nutritional outcomes, there is a need to describe the pathways through which these outcomes are achieved.

## Author contributions

JM, NM, BO, and ST designed and planned the study protocol, conducted database searches and screening, participated in data analysis, and drafted the manuscript. All authors planned the study protocol, and designed, revised, and approved the final manuscript.

## Footnotes

1
https://www.rayyan.ai/

2
https://training.cochrane.org/online-learning/core-software/revman
