# Supplementary material for: The impact of livestock interventions on nutritional outcomes of children younger than 5 years old and women in Africa: a systematic review and meta-analysis
Source: Front Nutr. 2023 Jul 6;10:1166495. doi: 10.3389/fnut.2023.1166495 (PMC10358768; doi:10.3389/fnut.2023.1166495)
Supplement: Supplementary file 1 [file Table_1.docx]

Supplementary Table 1: Summary of key livestock intervention studies on linkages between livestock interventions and nutritional outcomes among women and children <5 years of age.

| Study tile/Author(s), country | Study design | Livestock intervention/study objective | Main outcome measure(s) | Key qualitative findings | Key quantitative findings (effect sizes) |
| --- | --- | --- | --- | --- | --- |
| *Studies evaluating the impact of livestock-oriented programs* | | | | | |
| Got Milk? The Impact of Heifer international Livestock donations programs in Rwanda on nutritional outcomes  Rawlins et al., 2014, Rwanda. | Cross-sectional impact evaluation study in HHs with children < 5 years | Dairy cow and meat goat donation program | - Dietary diversity - Child anthropometry (weight, height/length) | - Increased individual dietary diversity for dairy cow beneficiary HHs. - Increased milk consumption for dairy cow beneficiaries and higher meat consumption for goat beneficiaries. | - Receiving a dairy cow was associated with an average increase of 1.17 food groups consumed - Marginally statistically significant reductions in WHZ z-scores and WAZ z-scores of approximately 0.4 SDs for meat goat recipients, and reductions in HAZ z-scores of approximately 0.5 SDs for dairy cow recipients. |
| Small-animal revolving funds: An innovative programming model to increase access to and consumption of animal-source foods by rural households in Malawi.  MacDonald et al., 2010, Malawi. | Cross-sectional surveys to evaluate program effectiveness | Distribution of small animals such as goats, rabbits, chickens, and guinea fowls to rural households accompanied by training on animal husbandry and intensive nutrition education to promote the consumption of animal products | Nutritional status and prevalence of anemia | Increased access to and consumption of ASFs for the intervention households. | - Egg consumption - increased from 28% at baseline to 52% at the end line - Chicken meat consumption increased from 33% to 58% and goat meat consumption from 13% to 26%. - Stunting reduced from 56% to 40%, underweight from 29% to 13%, and wasting from 8% to 2%. - Reduction in anemia prevalence in pregnant women from 59% to 48%. |
| Small-scale egg production centers increase children's egg consumption in rural Zambia.  Dumas et al., 2018, Zambia. | - Repeated cross-sectional design | Establishment of egg production centers – select farmers were given 40 layer hens. | - Children egg consumption - Children's nutritional status measured by stunting (HAZ) | - Increased egg consumption in the project area compared to the control - No impact on child HAZ | Significant increase in child egg consumption (OR 5.53 95% CI (2.90 - 10.58). |
| Enhancing the role of livestock production in improving the nutritional status of farming families: Lessons from a dairy goat development project in Eastern Ethiopia.  Kassa et al., 2003. Ethiopia. | - Cross-sectional survey for an intervention–control comparison | Donation of goats (crossbreeds and local breeds) to project beneficiaries in the dairy goat project by Farm Africa in Ethiopia compared to the control group | - Nutritional status of children < 5 years of age | - Increased dairy goat production was not accompanied by better utilization of foods of animal origin, especially milk. | - No statistically significant differences in the consumption of animal-source foods and nutritional status between the intervention and control groups |
| *Nutrition-sensitive value chain interventions* | | | | | |
| Delivery of iron-fortified yogurt, through a dairy value chain program, increases hemoglobin concentration among children 24 to 59 months old in Northern Senegal: A cluster randomized control trial.  Le port et al., 2017, Senegal. | - Cluster Randomized control trial (cRCT) | Provision of micronutrient-fortified yogurt (MNFY) and BCC to the intervention group compared to the control receiving only BCC.  Intervention group: received 1 sachet of MNFY per day for 7 days for each child aged 24-59 months.  Control group: received BCC only. messages on essential nutrition action (ENA) delivered through group sessions, home visits, community meetings, and radio spots). | - Child HB concentration and prevalence of anemia | - The nutrition-sensitive dairy value chain approach proved to be an effective way to improve Hb in preschool children. | - Non-significant decrease in anemia prevalence - Statistically significant greater increase in Hb (+0.55g/dl) in the intervention group compared to the control group; larger in boys (+0.72) than in girls (+0.38), not significant. |
| *Summary of evidence from observational studies* | | | | | |
| Cows, Missing Milk Markets, and Nutrition in Rural Ethiopia.  Hoddinott, Headey, and Dereje 2015, Ethiopia. | Cross-sectional | Test the association between cow ownership and child dietary intake and anthropometry comparing HHS that owns cows and those that do not. | - Dairy intake (7-day recall in children 6 - 24 months old). - Child (6-59 months old) anthropometry: HAZ, WHZ stunting. | - Cow ownership was associated with greater milk consumption, increased linear growth, and reduced stunting. | Cow ownership was associated with greater milk consumption, an increase in Height-for-age z-score (HAZ) scores of between 0.25 and 0.47 standard deviations, and reduced probability of stunting ranging from 6% to 13% for children 12 - 18 months old but no impact on WHZ |
| Does ownership of improved dairy cow breeds improve child nutrition? A pathway analysis for Uganda.  Kabunga et al., 2017, Uganda. | Cross-sectional analysis of data from Uganda 2009/2010 National Panel Survey (UNPS). | Breed improvement. Assess the association between the adoption of improved dairy cows with milk consumption and child anthropometry | - Nutritional outcome indicators (stunting (HAZ), wasting (WHZ), and underweight (WAZ)). - Milk yield, own-produced milk intake, and milk sales | - Improved dairy cow adoption is associated with increased milk consumption and reduced stunting (HAZ) but not with underweight (WAZ) and wasting (WHZ). | No quantitative data provided |
| Do low-income households in Tanzania derive income and nutrition benefits from dairy innovation  and dairy production? Kidoido and Korir 2015., Tanzania. | Cross-sectional analysis of Tanzania LSMS-ISA household panel data of 2008/2009 and 2010/2011. | Test the association between improved dairy production, household income, and child anthropometry | - Child nutritional status - Height-for-age (HAZ), Weight-for-height (WHZ), and Weight-for - age (WAZ) for children aged 0-60 months | - Dairy consumption improved child nutritional status (HAZ, WAZ, and WHZ) | - Dairy consumption was positively associated with HAZ, WAZ, and WHZ in low-income HHs |
| Is Exposure to Poultry Harmful to Child Nutrition? An Observational Analysis for Rural Ethiopia. Headey and Hirvonen 2016, Ethiopia | Cross-sectional exploratory analysis of observational data | Test the associations between household poultry ownership, exposure of children to poultry in the home, and child anthropometry [child height-for-age Z-scores (HAZ)]. | - Length or height-for-age Z-scores (HAZ) - Intermediate outcomes: Dietary diversity and exposure to diseases | - Poultry ownership was associated with improved child height-for-age Z scores (HAZ) | - Poultry ownership was positively associated with child HAZ [β = 0.291, SE = 0.094]. - Corralling poultry in the household dwelling overnight was negatively associated with HAZ [β =   -0.250, SE = 0.118]. |
| The Relationship between Livestock Ownership and Child Stunting in Three Countries in Eastern Africa Using National Survey Data. Mosites et al., 2015, Ethiopia, Kenya, and Uganda | Cross-sectional, analysis of Demographic and Health Survey (DHS) datasets from Ethiopia (2011), Kenya (2008–2009), and Uganda (2010). | Test association between livestock ownership and child stunting.  Compare stunting status across levels of livestock ownership. | - Stunting | - Livestock ownership was associated with reduced child stunting prevalence. | - Significant association between livestock ownership with lower stunting prevalence in Ethiopia (Prevalence Ratio [PR] 0.95, 95% CI 0.92–0.98) and Uganda (PR 0.87, 95% CI 0.79–0.97), but not in Kenya (PR 1.01, 95% CI 0.96–1.07). |
| Relationship between Household Livestock Ownership, Livestock Disease, and Young Child Growth.  Mosites et al., 2016, Kenya. | - Prospective cohort study. | Comparison of child growth (stunting) among households owning livestock and those that did not own any livestock.  Test association of livestock ownership and livestock disease, with child anthropometry. | - Stunting (HAZ), Wasting (WHZ), annualized child growth rate (cm/year), and mean monthly growth rate | - The study found no association between ownership of livestock and child growth status. However, disease episodes in household livestock may be related to a lower child growth rate in some groups | - Livestock ownership not associated with HAZ, WHZ, or growth rates - Livestock disease was associated with growth rates only in some months (June–November) and among children aged 0–23 months. |
| Camel milk consumption is associated with less childhood stunting and underweight than bovine milk in rural pastoral districts of Somali, Ethiopia: a cross-sectional study. Muleta et al., 2021,  Ethiopia | - Cross-sectional study | To compare the prevalence of growth failures between consumers of camel milk and bovine milk | - child nutritional status - height-for-age, weight-for-age, and weight-for-height z – scores. | - Camel milk consumption was associated with a lower prevalence of stunting and being underweight than bovine milk. | - Higher proportion of pre-schoolers consuming bovine milk were stunted (72 vs. 28 %; P < 0⋅001) and underweight (70⋅1 vs. 29⋅9 %; P < 0⋅001) compared with camel milk consumers, but not wasting - Severe stunting (76 vs. 24 %; P =0⋅002), severe wasting (66 vs. 34 %; P = 0⋅048), and severe underweight (78 vs. 22 %; P <0⋅001) were observed in bovine milk consumers |
| Associations between livestock ownership and lower odds of anemia among children aged 6–59 months are not mediated by animal-source food consumption in Ghana.  Lambrecht et al., 2021. Ghana | - Cross-sectional study with a comparison between households that owned livestock and households that did not own livestock | Assess the association between household livestock ownership and child anemia and examine whether this relationship is mediated by child ASF consumption or by child morbidity and inflammation | - Child anemia, defined as Hb < 11.0 g/dL for children aged 6–59 months | - Livestock ownership was associated with a reduced prevalence of anemia in children. - Consumption of ASFs did not mediate the observed association between livestock ownership and child anemia | - Children from households owning cattle, small livestock (goats, sheep, or pigs), and poultry, had lower odds of anemia compared to children from households that did not own livestock (OR [95% CI]:0.32 [0.14, 0.71]). |
| Camel milk consumption was associated with a lower prevalence of anemia among preschool children in rural pastoral districts of Somalia,  eastern Ethiopia. Muleta et al., 2021  Ethiopia | - Cross-sectional study | Children were selected from random households with lactating camels or cattle. | - Hemoglobin (Hb) | - Camel milk consumption was associated with a lower prevalence of anemia compared with the consumption of bovine milk. | - Anemia (Hb <11 g/dL) was found in 59.8% of the overall sample, whereas it was found in 42.7% and in 75.4% of CaM and BM consumers, respectively. - Children who consumed BM and had intestinal parasites were 3.1 and 3.3 times more likely to be anemic (aOR 3.12; 95% CI, 1.27-7.66) and (aOR,3.32; 95% CI, 1.39-7.91), respectively, than their counterparts. |
| Malnutrition among children in rural Malawian fish-farming households.  H. Aiga et al.,2009  Malawi | - Cross-sectional study | Compare the prevalence of stunting, underweight, and wasting among children aged 6—59 months between fish-farming and non-fish-farming households | - Prevalence of malnutrition (stunting, underweight, and wasting | - Overall, a lower prevalence of malnutrition was detected among the children in fish-farming households than those in non-fish-farming households in all the malnutrition indicators, i.e., stunting, underweight, and wasting | - Significant difference between fish-farming and non-fish farming households was confirmed only in the prevalence of severe underweight (<-3 WAZ) (P = 0.045) and global underweight (<-2 WAZ) (P = 0.042) parameters |
| Nutrition in agricultural development: Intensive dairy farming by rural smallholders. Hoorweg, Leegwater & Veerman, 2000  Kenya | - Cross-sectional study | Comparison of nutritional status among children from dairy farmers, dairy customers, and a rural population children group (not practicing dairy farming). | - Nutritional status (height-for-age, weight-for-age, and weight-for-height) | A positive relationship between milk consumption and the nutritional status of children, independent of household income, energy intake, and level of education was confirmed. | - Better nutritional status (height-for-age, weight-for-age, and weight-for-height) of preschool children among dairy farmers and dairy customers than in children from the rural population. |
| Smallholder milk market participation, dietary diversity, and nutritional status among young children in Ethiopia.  Lenjiso et al., 2016  Ethiopia. | Quasi-experimental analysis of survey data for households participating in smallholder milk markets and non-participants in children < 5 years | Comparison of nutritional status among children from households participating in smallholder milk market compared to non-participant households | - Dietary diversity - Child anthropometry (wasting, stunting & underweight) | - Young children from milk market participant households had significantly higher levels of dietary diversity - Better child nutritional status in milk market participant households - Milk market participation was associated with higher milk production and household income. | - Milk market participant households had a mean dietary diversity score of 5.3 while non-participant households had a score of 4.3. - Children from non-participant households had 11.3 % more likelihood to be wasted, 35% more likelihood to be stunted, and were 19.8% more likely to be underweight compared to children from milk market participant households. |
| *Summary of evidence from experimental studies* | | | | | |
| Meat and milk intakes and toddler growth: a comparison feeding intervention of animal-source foods in rural Kenya. Long et al., 2012  Kenya | Cluster randomized controlled trial | Comparison feeding intervention of three groups to examine the effect of ASFs on toddlers’ growth. Intervention groups included plain millet porridge (plain group), porridge with milk (milk group), and porridge with beef (meat group). Red meat in porridge (370 g/day). Milk in porridge (360 gr/day). Given 5 days/week for 5 months | - Linear growth (HAZ), MUAC, MAFA, MAMA - Anthropometry (MUAC) measurements | - Better linear growth in the milk group - Better nutrition status (MUAC) in the milk group than in the meat group | - Linear growth was significantly greater for the milk group than for the meat group (p=0.0025). - Slope of growth of the mid-arm muscle area of the plain group was significantly greater than in the meat group (p=0.0046). - Greater MUAC growth rate in the milk group than in the meat group (p=0.0418). |
| Effects of n–3 long-chain PUFA supplementation to lactating mothers  and their breastfed children on child growth and morbidity: a 2 X 2  factorial randomized controlled trial in rural Ethiopia. Argaw et al., 2018  Ethiopia. | Randomized, double-blind, placebo-controlled  trial | - Fish oil supplementation. Four different arms. - MCI - lactating mothers and children received fish oil intervention, - MI - lactating mother received fish oil supplementation and the child received placebo control. - CI - child received fish oil intervention and the mother received a placebo control.   C - both mother and child received a placebo supplement or control | - Linear growth - morbidity and systemic inflammation | Fish oil supplementation had no benefits on linear growth or morbidity. | - No significant intervention effect was found on linear growth, morbidity, or systemic inflammation. - Supplementation given directly to children moderately increased relative weight gain (effect size:0.022/mo 95% CI 0.005 - 0.039/mo) |
| An agriculture–nutrition intervention improved children's diet and growth in a randomized trial in Ghana.  Marquis et al.,2018  Ghana. | Cluster randomized controlled trial | Provision of chicken for egg production and training sessions on poultry production compared to the control group | - Dietary diversity - Child nutritional status (HAZ, WAZ, WHZ) - Consumption of eggs | Improved dietary diversity and better nutritional status for children in the intervention group | Compared to children in the control group, children in the intervention group met minimum diet diversity (AOR 1.65, 95% CI 1.02 - 2.69) and had higher LAZ/HAZ β=0.22 95% CI 0.09, 0.34) and WAZ (β=0.15 95% CI 0.00 - 0.30) |
| Impacts of an egg complementary feeding trial on energy intake and dietary diversity in Malawi.  Lutter et al., 2021  Malawi. | A randomized controlled trial | Provided an egg a day for 6 months or assigned to a control group | - Dietary diversity - Energy intake | - The intervention resulted in higher dietary diversity and an increased percentage of children attaining a minimum dietary diversity cut-off. | 80% of children in the egg group met minimum dietary diversity compared to 60% at the end-line in the control group  At the midline and end line, the usual energy intake from eggs was approximately 30 kcal/day higher in the egg group compared with controls (p < 0.0001   - Egg consumption was more than 9 times higher in the egg group than in the control group |
| The effect of bovine colostrum/egg supplementation compared with  corn/soy flour in young Malawian children: a randomized, controlled clinical trial. Bierut et al., 2021  Malawi | Prospective, randomized, blinded, placebo-controlled clinical trial | - The intervention group received a daily nutritional supplement of BC/egg, and the control group received an isoenergetic supplement of corn/soy flour from the ages of 9 to 12 months   Both groups received multiple micronutrients | - Primary outcomes were changes in length-for-age z-scores (Δ LAZ) | - Intervention associated with less linear growth faltering | - Reduced linear growth faltering in intervention group (difference = 0.12 z-scores; P = 0.0011) - Lower prevalence of stunting was observed in the intervention group (n = 47/137) compared to the control group (n = 62/127) at 17 months (RR = 0.70; 95% CI: 0.52, 0.94). |
| Animal Source Food Social and Behavior Change Communication Intervention Among Girinka Livestock Transfer Beneficiaries in Rwanda: A Cluster Randomized Evaluation. Flax et al., 2021  Rwanda. | A Cluster Randomized  trial | - SBCC intervention to promote the consumption of ASFs, especially cow milk, in households that had received a cow from the Girinka program. | - Dietary diversity -minimum dietary diversity (consumption of ≥4 food groups in the past 24 hours) - Milk consumption in the past 24 hours | - No significant differences between the intervention and control groups on diet diversity - Increased milk consumption in children in the intervention group compared to the control group | - Increased milk consumption in the intervention group compared to the control group (OR 2.1, 95%CI 1.1, 3.9) |
| Early Child Development Outcomes of a Randomized Trial Providing One Egg Per Day to Children Aged 6 to 15 Months in Malawi.  Prado et al., 2020  Malawi | Individually randomized controlled trial | - Provision of one egg per day for the intervention group child during twice-weekly home visits for 6 months. - The control group was visited twice per week and received messages about hygiene and handwashing during food preparation but did not receive eggs or any other foods during the study period. | Effect of the intervention on child development | The provision of one egg per day had no overall effect on child development in this population of children | Intervention and control groups did not significantly differ in any developmental score, with the  exception that a smaller percentage of children were delayed in fine motor development in the intervention group (10.6%) compared with the control group (16.5%; prevalence ratio: 0.59, 95% CI: 0.38–0.91). |
| A Chicken Production Intervention and Additional Nutrition Behaviour Change Component Increased Child Growth in Ethiopia: A Cluster-Randomized Trial.  Passarelli et al.,2020. Ethiopia | - A cluster randomized control trial | Provision of genetically improved chicken and nutrition-sensitive behavior change communication. The control group received no intervention. | - Height-for-age z scores (HAZ), weight-for-age z scores (WAZ), and weight-for-height z scores (WHZ) | - The intervention improved HAZ and WAZ compared to the control - Improved dietary diversity | The intervention group had higher HAZ (MD: 0.28; 95% CI: 0.05, 0.50) and WAZ (MD: 0.18; 95% CI: 0.01, 0.36) compared to the control but it was not statistically significant. |
| Burkina Faso.  Behavior Change, Egg Consumption, and Child Nutrition: A Cluster Randomized Controlled Trial.  McKune et al., 2020. | Cluster randomized controlled trial | - Each child in the full intervention arm received four chickens (three gifted by a community champion & one from the child’s family), and mothers received the 10-month behavior change package - Participants in the partial intervention arm received only the behavior change package | - Primary outcome: child egg consumption - Secondary outcome: poultry production, women empowerment, and anthropometric indices | - Both full and partial interventions significantly increased egg consumption compared to the control group - The intervention had no statistically significant effect on child stunting | - Full intervention significantly increased poultry production (β= 11.6; 95% CI 8.3–15; P =1.1 3 1025) and women’s decision-making about eggs (β = .66; P = .02), and significantly decreased wasting ((β = .58; P = .03) and underweight ((β = .47; P =0.02). |
| The effect of eggs on early child growth in rural Malawi: The Mazira Project randomized controlled trial.  Stewart et al., 2019  Malawi | Individually randomized controlled trial | Provision of one egg per day or assigned to the control group.  Eggs were provided during twice-weekly home visits for 6 months. Control households were visited with the same frequency | Child linear growth | - Increased egg consumption in the intervention group compared to the control group - The provision of one egg per day to children in rural Malawi had no overall effect on linear growth | - No intervention effect on length-for-age, weight-for-age, or weight-for-length z scores - Significantly higher head circumference for age z score of 0.18 (95% CI: 0.01, 0.34) in the intervention group compared to the control group. |
| Impacts of an egg intervention on nutrient adequacy among young Malawian children. Caswell et al., 2021  Malawi. | A randomized controlled trial | Provision of an egg per day to children aged 6 – 15 months | - Nutrient intake adequacy and micronutrient density. | - Higher Nutrient intake adequacy and micronutrient density in the intervention group. |  |
| Vaccination of household chickens results in a shift in young children’s diet and improves child growth in rural Kenya.  Otiang et al 2022  Kenya | Randomized controlled trial | Quarterly vaccination of chickens against Newcastle Disease Virus (NDV) plus parasite control for the intervention arm while the control arm received parasite control only | - Consumption of ASFs - Child growth | - The intervention increased the consumption of ASFs and improved children’s HAZ and WHZ | - Increased consumption of ASFs (24% increase) by children in the intervention households compared to the control group - Overall increase in both HAZ and WHZ z-scores in the intervention group relative to the control group. |

HHs – households; HB – hemoglobin; cRCT – cluster randomized controlled trial; BCC – behavior change communication; EPCs – egg production centers; ASFs – animal source foods; WHZs – weight–for–height z-scores; WAZs – weight–for–age z-scores and HAZs – height-for-age z-scores.
